# A PARP1-Erk2 synergism is required for stimulation-induced expression of immediate early genes

**Published:** 2016-07-11

**Authors:** M. Cohen-Armon

**Affiliations:** Department of Physiology and Pharmacology, Sackler School of Medicine, and the Sagol School of Neuroscience, Tel-Aviv University, Tel-Aviv, 69978, Israel

**Keywords:** Immediate early genes, PARP1-Erk2 synergism, LTP generation

## Abstract

A PARP1-Erk2 synergism was required to generate synaptic long-term potentiation in the CA3-CA1 hippocampal connections. This molecular mechanism was associated with the recently identified pivotal role of polyADP-ribosylation in learning. High frequency electrical stimulation of cortical and hippocampal neurons induced binding of phosphorylated Erk2 (transported into the nucleus) to the nuclear protein PARP1. PARP1-Erk2 binding induced PARP1 activation and polyADP-ribosylation of its prominent substrate, linker histone H1. A facilitated access of PARP1-bound phosphorylated Erk2 to its substrates, transcription factors Elk1 and CREB was attributed to the release of polyADP-ribosylated H1 from the DNA, causing local DNA relaxation. Erk-induced phosphorylation of transcription factors activating the HAT activity of CBP (CREB binding protein), recruited acetylated histone H4 to the promoters of immediate early genes (IEG) *cfos, zif268* and *arc*, which are implicated in synaptic plasticity. In accordance, their induced expression was suppressed after PARP1 genetic deletion in PARP1-KO mice, or after PARP1 inhibition or silencing. Moreover, under these conditions, long-term synaptic potentiation (LTP) (indicating synaptic plasticity) was not generation in the hippocampal CA3-CA1 connections, and learning abilities were impaired. Furthermore, both IEG expression and LTP generation failed when cerebral neurons accumulated single strand DNA breaks, due to a predominant binding of PARP1 to nicked DNA, occluding its Erk binding sites. Thus, a declined synaptic plasticity is anticipated when aged cerebral neurons accumulate DNA single-strand breaks during life span.

PolyADP-ribose polymerases (PARPs) catalyze an abundant post-translational modification of proteins by polyADP-ribosylation. In this energy consuming protein modification, NAD (Nicotinamide adenine dinucleotide) derived ADP-ribosyl moieties form ADP-ribose polymers on glutamate, lysine and aspartate residues of PARPs and their substrates ^[1, 2]^. Binding of the most abundant nuclear protein PARP1 to DNA single-strand breaks activates the protein, and thereby promotes single strand DNA base-excision repair ^[1, 2]^.

Recent findings implicated PARP1 in additional mechanisms in the chromatin, not necessarily involving repair of damaged DNA ^[2–8]^. Moreover, mechanisms causing PARP1 activation in the absence of DNA were identified in cell-free systems ^[7, 9]^.

Numerous findings implicated the phosphorylation of extracellular signal regulated kinase-2 (Erk2) in synaptic plasticity and long-term memory ^[10–14]^. Interestingly, recent *in-vivo* experiments also revealed a pivotal role of PARP1 activation in long-term memory acquisition during learning ^[15–19]^, but the explicit molecular mechanism underlying this un-expected role of PARP1 was not identified.

By combining electrophysiological measurements with biochemical and structural biology methods, we identified a molecular mechanism in the chromatin of cerebral neurons, which is necessary for stimulation induced immediate early gene (IEG) expression implicated in synaptic plasticity ^[20]^. Long-term synaptic potentiation (LTP) has been associated with synaptic plasticity and long-term memory acquisition ^[21, 22]^.

The hippocampus plays an important role in forming and retaining new memories ^[21, 22]^. In our experiments, field excitatory postsynaptic potentials (fEPSPs) were recorded from stimulated CA3-CA1 connections in hippocampal slices of WT mice. High frequency electrical stimulation (100 Hz, 1s) induced synaptic long term potentiation (LTP) ^[20, 22, 23]^. LTP in the hippocampal CA3-CA1 connections is currently acceptable as a cellular model for long-term memory ^[21–23]^. LTP was not generated after PARP1 genetic deletion in the hippocampal CA3-CA1 connections of PARP1-KO mice, or after PARP1 inhibition ^[20]^.

Stimulation inducing LTP is restricted to a small subset of afferents in the hippocampus, which are impossible to identify and isolate ^[21, 23]^. In an attempt to identify molecular mechanisms associated with LTP, we used a model system of similarly stimulated cultured cortical and hippocampal neurons. These cerebral neurons were stimulated by a variety of stimulations including electrical stimulation of various frequencies ^[20]^. A high frequency electrical stimulation (3 repeats of a 100 Hz, 1 sec duration pulse, followed by a 10 sec pause) causes synaptic potentiation ^[20, 24, 25]^.

Notably, this high frequency stimulation induced expression of immediate early genes *c-fos, zif268* and *arc* in the cultured cerebral neurons (Figure 1) ^[20]^. The expression of theses IEG has been implicated in synaptic plasticity ^[26–30]^. The expression of *arc* lagged after *zif268* expression, probably due to Zif268 (Egr1) acting as one of *arc* transcription factors (Figure 1) ^[28]^. The applied high frequency stimulation did not induce a non-specific Erk-dependent gene expression ^[20]^.

The expression of *c-fos, zif268* and *arc* was suppressed in cerebral neurons treated with PARP inhibitors, as well as after PARP1 silencing (by siRNA, 150 nM, 72 hours) or its genetic deletion in cerebral neurons of PARP1-KO mice ^[20]^. These results suggested a possible implication of PARP1 in stimulation-induced expression of *c-fos, zif268* and *arc*.

A possible role of PARP1 activation in the recruitment of RNA-Pol-II and transcription factors to the IEG promoters ^[31]^ seemed unlikely, in-view of recent evidence indicating poised RNA-Pol-II in the IEG promoters ^[32]^, and transcription factors of the IEG (Elk1, CREB) bound to HAT (histone acetyl-transferase) ^[33, 34]^. Instead, we examined a possible role of PARP1 in Erk-induced phosphorylation of transcription factors CREB and Elk1. Their phosphorylation induces the HAT activity of CBP (CREB binding protein) promoting gene expression implicated in long-term memory ^[35]^.

Phosphorylated Erk1/2 is translocated into the nucleus ^[36, 37]^. Co-immunoprecipitation of phosphorylated Erk2 with PARP1 was measured in the chromatin of electrically stimulated cerebral neurons by high frequency stimulation ^[20]^. Concomitantly, Erk-bound PARP1 and its prominent substrate linker histone H1 became highly polyADP-ribosylated ^[20]^. Notably, PARP1 was not similarly activated in un-stimulated neurons nor in neurons stimulated by low frequency stimulations ^[20]^.

Erk-induced PARP1 polyADP-ribosylation could be attributed to intra-molecular calculated movements in PARP1 bound to phosphorylated Erk2, exposing the NAD binding site in its catalytic domain ^[20]^ (according to cell-free experiments one PARP1 binds two molecules of phosphorylated Erk2^9^). Here, bioinformatics calculation complied with polyADP-ribosylation of Erk-bound PARP1 even at low NAD concentrations ^[9,20]^. At low [^32^P]NAD concentrations, Erk-induced [^32^P]polyADP-ribosylation of recombinant PARP1 bound to recombinant phosphorylated Erk2 was higher than the [^32^P]polyADP- ribosylation of recombinant PARP1 bound to nicked DNA ^[9, 20]^.

A possible role of this Erk-induced PARP1 activation in stimulation-induced IEG expression was examined by measuring PARP1-dependent recruitment of phosphorylated Erk2 to the promoters of *c-fos* and *zif268* ^[20]^. The ChIP (chromatin immunoprecipitation) assay was used to identify recruited proteins to DNA segments in the promoters of the immediate early genes, in cultured cerebral neurons stimulated by brief high frequency electrical stimulation. Phosphorylated Erk2 and acetylated histone H4 co-immunoprecipitated with DNA segments in the promoters of *c-fos* and *zif268* in the stimulated cerebral neurons of WT mice. However, they were hardly bound to their promoters after PARP1 inhibition, or PARP1 genetic deletion in stimulated cerebral neurons of PARP1-KO mice ^[20]^. These results associated polyADP-ribosylation of PARP1 bound to phosphorylated Erk2 with PARP1-dependent recruitment of phosphorylated Erk2 and acetylated H4 to the promoters of *cfos* and *zif268.*

Furthermore, polyADP-ribosylation prevented PARP1 binding to its substrate linker histone H1 ^[20]^, associating Erk-induced polyADP-ribosylation of PARP1 and linker histone H1 with H1 release causing a local DNA relaxation ^[38]^. This local H1 polyADP-ribosylation could render CREB and Elk1 accessible to PARP1-bound phosphorylated Erk2. Phosphorylation of transcription factors Elk1 and CREB, inducing the HAT activity of CBP ^[33, 39]^ complied with the recruitment of acetylated H4 to promoters of *cfos* and *zif268* and their expression in response to stimulation ^[20]^.

Notably, molecular modifications in DNA-bound PARP1 occlude the binding sites of phosphorylated Erk in the aa556-1014 domain of PARP1. Their occlusion could prevent PARP1 binding to phosphorylated Erk2 in response to stimulation ^[20, 40, 41]^. This finding anticipates an interference of single-strand DNA breaks with PARP1 binding to phosphorylated Erk2 and IEG expression ^[20]^.

The effect of nicked DNA on IEG expression was examined in cerebral neurons of PARP1 KO mice transfected with either PARP1 or truncated PARP1 constructs ^[20]^. The predominant binding of PARP1 to single strand DNA breaks interfered with c*fos* and *zif268* expression only when cerebral neurons of PARP1-KO mice were transfected with GFP-fusion vectors encoding full length PARP1.

A low expression of *c-fos* and *zif268* was measured in stimulated cerebral neurons of PARP1-KO mice. However, these IEG were expressed in stimulated cerebral neurons of PARP1-KO mice transfected with PARP1 constructs encoding PARP1 domains containing Erk binding sites. Their expression was impaired after insertion of single strand DNA breaks in PARP-KO cerebral neurons transfected with full length PARP1 containing its DNA-binding domain. DNA single-strand breaks did not interfere with the expression of *cfos* and *zif268* in PARP-KO cerebral neurons transfected with truncated PARP1 lacking its DNA binding domain ^[20]^. These findings indicated an interference of PARP1-binding to nicked DNA with IEG expression.

In compliance, low amounts of proteins/transcription factors c-Fos, Zif and Arc were measured in response to stimulation of cerebral neurons with nicked DNA. Similarly, the exposure of cerebral neurons to hypoxia, causing DNA single-strand breaks down-regulated stimulation-induced *cfos* and *zif268* expression ^[20]^. Since, hypoxia interferes with synaptic plasticity in the hippocampus ^[42]^, possible effects of single-strand DNA breaks on the generation of LTP were examined ^[20]^.

Binding of phosphorylated Erk2 to PARP1 was measured in cell nuclei of stimulated hippocampal neurons of WT mice briefly stimulated by high frequency (100 Hz, 1s) stimulation. Treatment causing single strand DNA breaks in these neurons interfered with PARP1-Erk2 binding.

Notably, LTP failed to generate in stimulated hippocampal CA3-CA1 connections after treatment inducing single-strand breaks. However, already generated LTP was not impaired by inducing DNA single-strand breaks, similarly to the maintenance of LTP generated before application of PARP1 or MEK inhibitors ^[20]^. These results supported the notion of PARP1-Erk2 synergism required for LTP generation- not maintenance.

In compliance, IEG expression was scarcely affected in the presence of nicked DNA when cerebral neurons were pre-treated with the PARG (polyADP-ribose glycohydrolase) inhibitor gallotannin ^[20, 43]^, which prevents a recurrent binding of activated PARP1 to the negatively charged DNA ^[43]^. Since PARP1 polyADP-ribosylation did not interfere with PARP1-Erk2 binding, PARG inhibition could preserve PARP1-Erk2 binding and IEG expression in the presence of single-strand DNA breaks by preventing the binding of polyADP-ribosylated PARP1 to DNA. This hypothesis was confirmed in a cell-free system by measuring the dose-dependent effect of recombinant PARP1 polyADP-ribosylation on its binding to recombinant phosphorylated Erk2 in the presence of nicked DNA ^[20]^.

Thus, the indicated failure to generate LTP due to accumulating DNA single-strand breaks in aged cerebral neurons could be associated with the deterioration of memory acquisition and learning abilities, frequently experienced in senescence ^[44, 45]^.

The DNA of the irreplaceable mammalian cerebral neurons is constantly exposed to reactive oxygen species (ROS), normally produced in their mitochondria due to high-energy demands in neurons of the central nervous system. ROS cause single strand DNA breaks ^[46, 47]^. A constant exposure to these DNA damaging reactions produce single strand breaks accumulating during life span, despite the existing DNA repair mechanisms ^[45–47]^. As a result, IEG expression implicated in synaptic plasticity could be impaired in aged cerebral neurons due to the predominant binding of PARP1 to accumulated nicks in their DNA ^[20]^. In support, recent evidence indicated an improved long-term memory acquisition in aged mice treated with the PARG inhibitor gallotannin ^[48]^.

In summary, these results disclosed a molecular mechanism in the chromatin, linking Erk2-induced PARP1 polyADP-ribosylation with Erk2-induced phosphorylation of IEG transcription factors, required for stimulation induced IEG expression implicated in synaptic plasticity ^[20]^. This molecular mechanism was manipulated by agents affecting PARP1-Erk binding and synergism. In trained animals PARP1 inhibitors prevented long-term memory acquisition during learning without erasing past memory ^[15, 17]^.

## Figures and Tables

**Figure 1:**
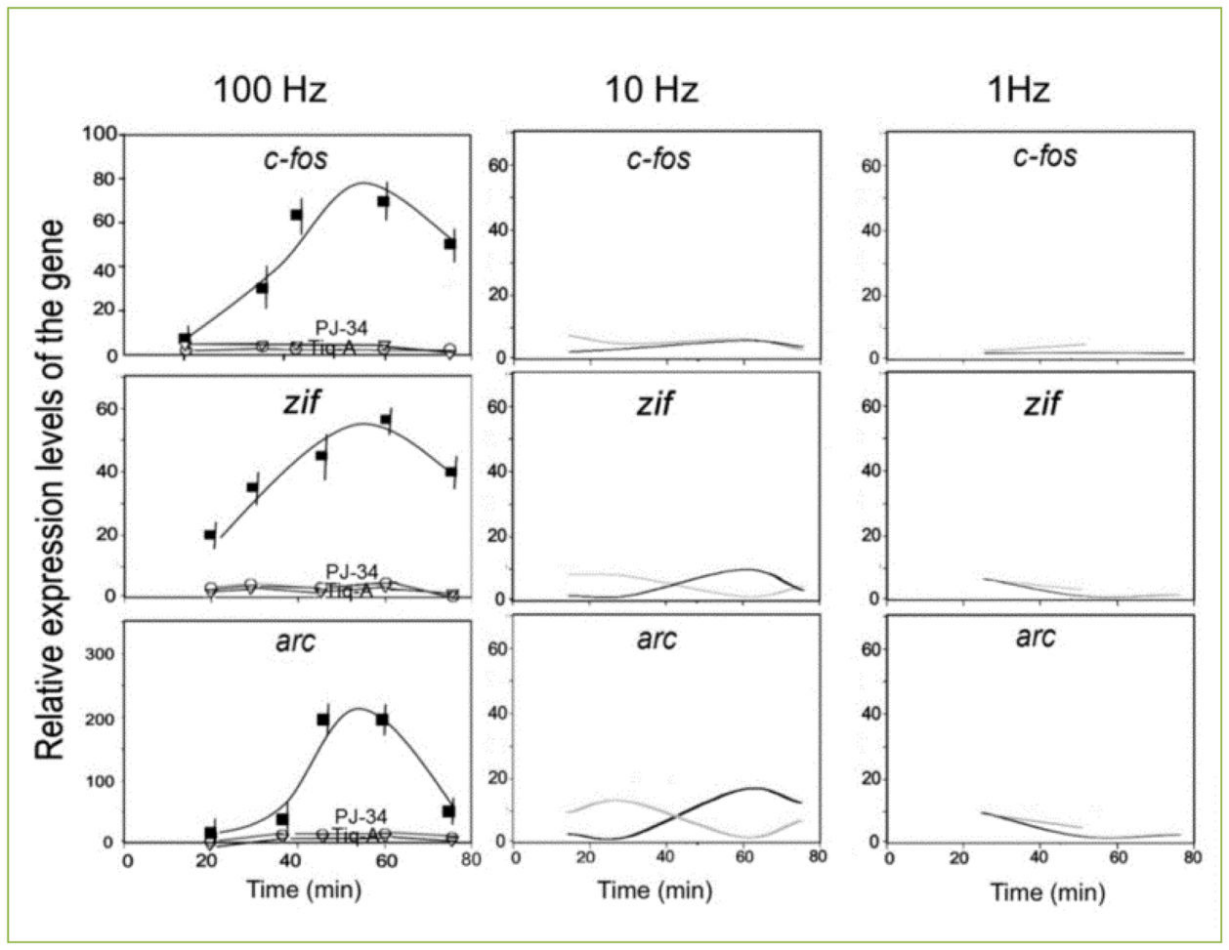
PARP1 mediated expression of immediate early genes *cfos, zif268* and *arc* in response to stimulation of cultured cerebral neurons The relative expression rate of immediate early genes *c-fos, zif268* and *arc* was measured by RT-PCR at the indicated time intervals after stimulation of cultured rat cerebral neurons (3 repeats of 100 Hz, 10 Hz or 1 Hz stimulation, 1 sec duration, each followed by 10 sec pause). An enhanced expression rate of *c-fos, zif268* and *arc* was measured only in response to the high frequency stimulation (100 Hz; black line). The stimulation-induced gene expression was suppressed after PARP inhibition with PJ-34 (10 μM) or Tiq-A (50 μM) (grey lines). Each value represents the mean value with calculated variation coefficient (Standard deviation divided by the average value) of 4 separate reactions in each of 4 experiments. Reprinted with permission [20]

## Abbreviations

IEG: immediate early genes
LTP: long-term potentiation
PARP: polyADP-ribose polymerase
Erk: extracellular signal regulated kinase
